# Assessing the Impact of a Hilly Environment on Depressive Symptoms among Community-Dwelling Older Adults in Japan: A Cross-Sectional Study

**DOI:** 10.3390/ijerph18094520

**Published:** 2021-04-24

**Authors:** Takafumi Abe, Kenta Okuyama, Tsuyoshi Hamano, Miwako Takeda, Masayuki Yamasaki, Minoru Isomura, Kunihiko Nakano, Kristina Sundquist, Toru Nabika

**Affiliations:** 1 Center for Community-Based Healthcare Research and Education (CoHRE), Head Office for Research and Academic Information, Shimane University, Shimane 693-8501, Japan; kenta.okuyama@med.lu.se (K.O.); thamano@cc.kyoto-su.ac.jp (T.H.); cohre1@med.shimane-u.ac.jp (M.T.); myamasak@hmn.shimane-u.ac.jp (M.Y.); isomura@hmn.shimane-u.ac.jp (M.I.); k-nakano@riko.shimane-u.ac.jp (K.N.); kristina.sundquist@med.lu.se (K.S.); 2 Center for Primary Health Care Research, Department of Clinical Sciences Malmö, Lund University, 20502 Malmö, Sweden; 3 Department of Sports Sociology and Health Sciences, Faculty of Sociology, Kyoto Sangyo University, Kyoto 603-8555, Japan; 4 Faculty of Human Sciences, Shimane University, Shimane 690-8504, Japan; 5 Department of Family Medicine and Community Health, Department of Population Health Science and Policy, Icahn School of Medicine at Mount Sinai, New York, NY 10029-5674, USA; 6 Department of Functional Pathology, Faculty of Medicine, Shimane University, Shimane 693-8501, Japan; nabika@med.shimane-u.ac.jp

**Keywords:** land slope, rural area, depression, public health

## Abstract

Although some neighborhood environmental factors have been found to affect depressive symptoms, few studies have focused on the impact of living in a hilly environment, i.e., land slope, on depressive symptoms among rural older adults. This cross-sectional study aimed to investigate whether a land slope is associated with depressive symptoms among older adults living in rural areas. Data were collected from 935 participants, aged 65 years and older, who lived in Shimane prefecture, Japan. Depressive symptoms were assessed using the Zung Self-Rating Depression Scale (SDS) and defined on the basis of an SDS score ≥ 40. Land slopes within a 400 m network buffer were assessed using geographic information systems. Odds ratios (ORs) with 95% confidence intervals (CIs) of depressive symptoms were estimated using logistic regression. A total of 215 (23.0%) participants reported depressive symptoms. The land slope was positively associated with depressive symptoms (OR = 1.04; 95% CI = 1.01–1.08) after adjusting for all confounders. In a rural setting, living in a hillier environment was associated with depressive symptoms among community-dwelling older adults in Japan.

## 1. Introduction

Depression is common among older adults [1]. The World Health Organization reported that depression is a leading disease that contributes to the burden of mental health [2]. The prevalence of depression in the global population was estimated to be 4.4% in 2015, peaking in older adulthood (above 7.5% among women aged 55–74 years, and above 5.5% among men) [3]. In Japan, the prevalence of depression was estimated to be 2.9% among older adults aged 60–89 years in 2019 [4]. As it is known, depression is a risk factor for cardiovascular disease, stroke, suicide, and mortality [5,6,7,8]. Therefore, preventing depressive symptoms among older adults is important within the field of public health.

Socio-demographic and modifiable variables (e.g., age, sex, educational level, sleep problems, physical activity, sedentary behavior, and lower back pain) were reported to be independently associated with depressive symptoms [9,10,11,12,13]. Recently, a systematic review found that social and physical neighborhood environments may predict depressive symptoms in older adults [14]. This meta-analysis indicated that neighborhood socio-economic status, collective efficacy, and personal/crime-related safety are negatively associated with depression outcomes. However, the previous studies included in this review were mostly based on urban settings. In addition, this review suggested that further studies are needed to examine associations between physical environment attributes and depression due to a lack of studies examining other neighborhood environmental factors [14].

Japan’s topography is quite different from that of several Western countries [15]. Specifically, hilly environments are the most typical feature of rural Japan [15]. For instance, Japan’s land area is predominantly (72.8%) mountainous or hilly, with the proportion of flat land (less than 3.0° land slope) accounting for only 14% of the total land area [16]. Thus, rural residents walk up and down steep slopes daily, which can increase the mechanical load on their knees [17]. Excessive physical loads may also lead to musculoskeletal diseases, such as knee and lower back pain, and this, in turn, can affect depressive symptoms by reducing one’s sleep quality [10,18]. In contrast, previous studies reported that steeper slopes had a protective effect against diabetes [19,20]. Residents who exercise more going up and down steep slopes might have better general health than those who do not. Hence, it is possible that there exists a relationship between living in a hilly rural environment and depressive symptoms. Although a previous study conducted in an urban area found that a hilly environment was positively associated with depressive symptoms in older women [21], no study has been conducted in a rural setting. Therefore, in the present study, we investigated whether land slopes were associated with depressive symptoms in older Japanese adults living in rural areas.

## 2. Materials and Methods

### 2.1. Study Design and Setting

**Figure 1:**
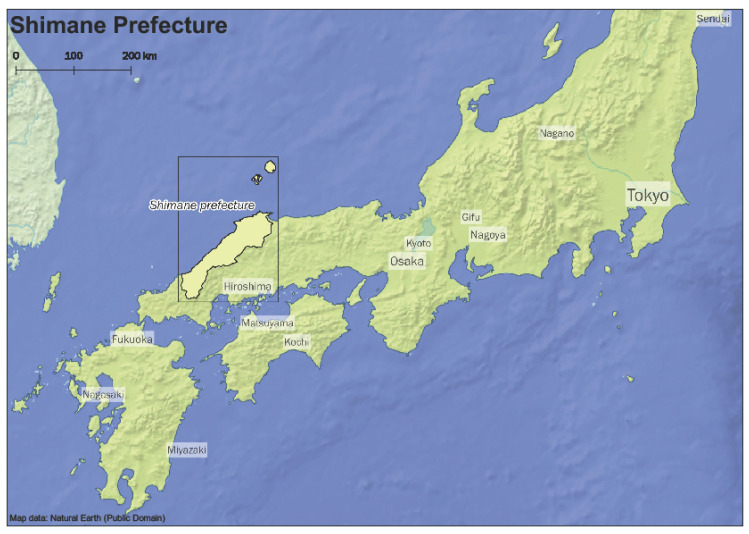
Study area: Shimane prefecture in Japan.

**Figure 2:**
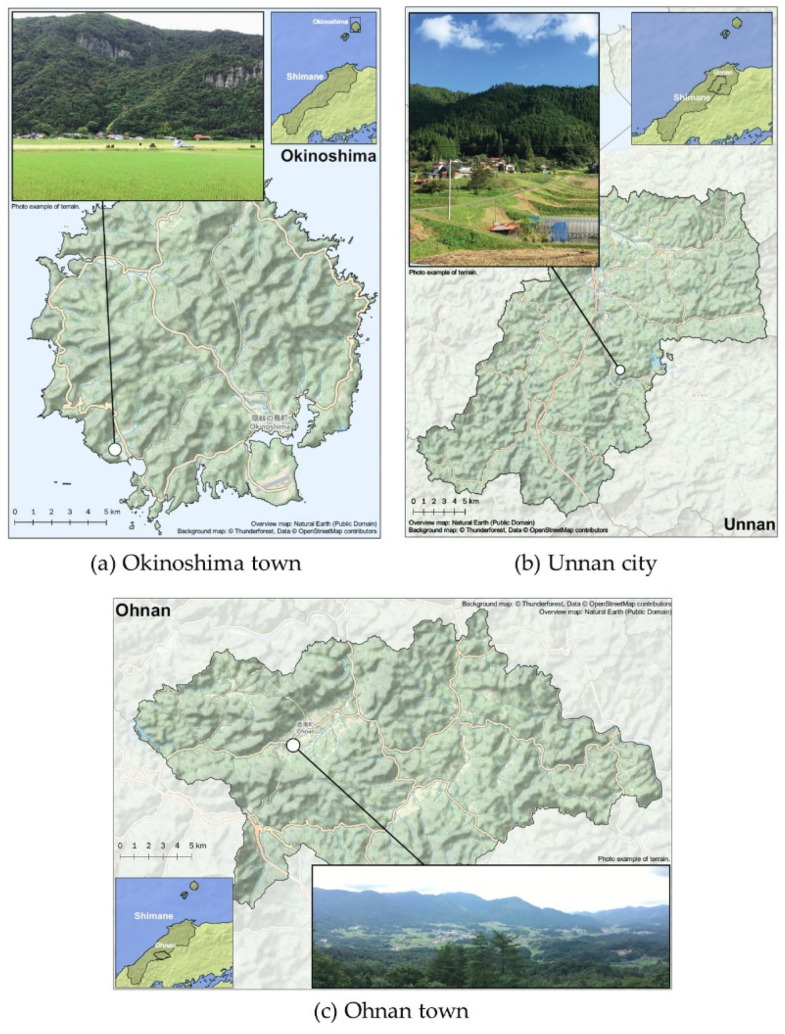
Geographical features of three municipalities. (**a**) Okinoshima town. (**b**) Unnan city. (**c**) Ohnan town.

This cross-sectional study was part of the Shimane CoHRE Study that was conducted by Shimane University in collaboration with an annual health checkup program in Okinoshima town, Unnan city, and Ohnan town (population density: 60.2, 70.6, and 26.5 person/km^2^, respectively; data from census in 2015), Shimane prefecture (Figure 1), Japan. These three municipalities are rural areas of Japan, as shown in Figure 2.

This study used health checkup data collected by a government agency in the three municipalities from June to November 2012. An annual health checkup is available once a year for residents aged 40 years and over who are covered by the National Health Insurance. The health checkup protocol has been recommended by the Japanese Ministry of Health, Labor and Welfare [22]. These data included an additional health survey collected from the same participants in the health checkup.

### 2.2. Ethical Considerations

We explained the research objectives of this study to the participants verbally before the health checkups. Written informed consent was obtained from all participants. The study protocol was approved by the Ethics Committee of Shimane University (#2888).

### 2.3. Participants

Inclusion criteria were older adults aged 65 years or above, who resided in the three municipalities and participated in health checkups. Exclusion criteria were participants with missing data on exposure, outcome, and confounders for statistical analyses. A total of 1551 older adults participated in the study. We excluded the data of 616 participants for the main statistical analyses due to missing data (i.e., depressive symptoms, *n* = 19; moderate to vigorous physical activity (MVPA), and sedentary time, *n* = 93; lower back pain, *n* = 2; years of education, *n* = 495; geographic information system (GIS) code, *n* = 7). Data from 935 participants were available for statistical analyses. There were no significant differences between participants with and without missing data in terms of sex (*p* = 0.29), age (*p* = 0.84), body mass index (BMI) (*p* = 0.09), and depressive symptoms (*p* = 0.27). 

### 2.4. Outcome Variable

Depressive symptoms were assessed using the Zung Self-Rating Depression Scale (SDS), a 20-item self-report questionnaire [23]. Each item is scored from 1 to 4, with a total score range of 20–80; higher scores indicate more severe depressive symptoms. A Japanese version of the SDS has been developed [24], and the test–retest reliability over seven days for each item was acceptable (*r* > 0.60). In a validation study [24], a chi-square analysis showed a statistically significant difference for each item between healthy participants and patients with depression (*p* < 0.05). Moreover, the total SDS score among the patient samples was significantly higher than that of the comparison sample (*p* < 0.01). In this study, a cut-off point (SDS score ≥ 40) was used to define the presence of depressive symptoms, as described in previous studies [24,25].

### 2.5. Exposure Variable

The land slope within a 400 m network buffer from each residential point for each participant was used as the main exposure variable. Land slope values (degree) were analyzed using ArcGIS 10.0 (Esri Inc., Redland, CA, USA) as follows. The network buffer is a polygonal geometric space that approximates the daily activity space of each participant along with the actual street network. The 400 m network buffer was found to be an appropriate activity space for people in a previous neighborhood study [26]. The buffer zone was computed along with the actual street network, which excluded non-habitable areas, such as forests, rivers, and mountains. Mean land slope was calculated within the 400 m network buffer as a degree in the angular unit for each resident. The data of mean land slope were created in 2011, stored in 5th mesh data (50:50 m grid), and obtained from the National Land Numerical Information, administered by the National Land Information Division, National Spatial Planning, and Regional Policy Bureau of Japan [27].

### 2.6. Covariates

The respondents’ sex (male or female), age (65–74 years, or 75 years and above, calculated using the respondent’s birthdate), current alcohol drinking status (yes or no), current smoking status (yes or no), getting enough sleep (yes or no), and residential area (Okinoshima town, Ohnan town, or Unnan city) were collected as part of the health checkups. Lower back pain (yes or no) [28], educational years (higher or lower than high school graduate: ≥12 years or <12 years) [29], MVPA, and sedentary time were examined using a self-reported questionnaire, the validated Japanese short version of the International Physical Activity Questionnaire [30,31]. We divided physical activity levels into two categories (at least ≥150 min/week or <150 min/week) using a cut-off value for metabolic equivalents (METs) (≥8.25 METs/week) based on the current MVPA recommendations [32] and systematic review [33]. Sedentary time was divided into two categories (<3 h/day or ≥3 h/day) via a median split. Height and weight were objectively assessed as part of a health checkup. BMI was calculated from participants’ height and weight (kg/m^2^) and divided into three categories using Asian cut-off points (underweight: <18.5; normal weight: 18.5–22.9; overweight: ≥23.0 kg/m^2^) [34].

### 2.7. Statistical Analyses

Descriptive statistics were calculated to assess differences in depressive symptoms as a function of demographic characteristics using chi-square tests for categorical data and the Mann–Whitney U test for continuous data.

The main analyses in this study used two models (a crude and an adjusted model) to examine whether land slope as the main exposure variable was associated with depressive symptoms. Binary logistic regressions were calculated to estimate odds ratios (ORs) and 95% confidence intervals (CIs) for those who experienced depressive symptoms from the land slope as a continuous variable. For the crude model, the analysis was conducted without any adjustments. For the adjusted model, the analysis was adjusted for sex (reference group = males), age (reference group = 65–74 years old), BMI (reference group = normal weight), current smoking status (reference group = no), current alcohol drinking status (reference group = no), physical activity (reference group = ≥150 min/week), sedentary time (reference group = <3 h/day), getting enough sleep (reference group = yes), lower back pain (reference group = no), educational years (reference group = ≥12 years), and residential area (reference group = Okinoshima town). All statistical analyses were performed using IBM SPSS Statistics 24.0 for Windows (IBM Corp., Armonk, NY, USA). For all analyses, *p*-values less than 0.05 were considered statistically significant. 

## 3. Results

Table 1 shows the differences in depressive symptoms based on participant characteristics. Of the 935 participants, 215 (23.0%) reported depressive symptoms. The prevalence of depressive symptoms significantly differed based on getting enough sleep (“no” = 46.2% vs. “yes” = 17.2%, *p* < 0.01) and lower back pain (“no” = 17.8% vs. “yes” = 28.6%, *p* < 0.01). The land slope was greater among participants with depressive symptoms (*p* = 0.01). There were no significant differences in depressive symptoms by respondents’ sex, age, BMI, current smoking status, current alcohol drinking status, physical activity, sedentary time, educational years, or residential area.

Table 2 shows the associations between land slope and depressive symptoms. Land slope was significantly associated with depressive symptoms in the crude (OR = 1.04 (95% CI, 1.01–1.08), *p* = 0.01) and adjusted models (OR = 1.04 (95% CI, 1.01–1.08), *p* = 0.02). In the adjusted model, older adults lacking sleep and those suffering from lower back pain had significantly higher odds of experiencing depressive symptoms (OR = 4.24 (95% CI, 2.94–6.13), *p* < 0.01; OR = 1.66 (95% CI, 1.19–2.30), *p* < 0.01, respectively). No significant associations were found for respondents’ sex, age, BMI, current smoking status, current alcohol drinking status, physical activity, sedentary time, educational years, or residential area. In both the models, the Hosmer–Lemeshow test was not significant (*p* > 0.293).

## 4. Discussion

This study is the first to examine whether land slope is related to depressive symptoms in older Japanese adults living in rural areas. We found that a greater land slope was significantly associated with depressive symptoms after adjusting for all confounders. The association between depressive symptoms with lack of sleep and lower back pain as individual factors was also shown. Our findings are consistent with a previous study [21]; Tanaka et al. reported that older women living in sloped areas had higher depression levels than individuals residing in non-sloped areas in urban Japan [21]. In this previous study [21], sloped and non-sloped areas were defined as more or less than five degrees, respectively. Our study indicated that the median of the slope degree was 9.22°. Thus, our study area included a higher slope degree than the urban area in the previous one. These findings represent valuable knowledge in that consideration of land slope is important in the formulation of prevention strategies for depression in rural areas of Japan. For example, health education is needed for older adults living around greater slopes to promote knowledge of depressive symptom prevention in rural areas, because physical neighborhood environments might not be easy to change. Our findings also provide information for the prevention of depressive symptoms among older adults through prevention and treatment of lack of sleep and lower back pain as individual factors.

To date, several studies have shown associations between slope and health outcomes. Using an ecological study design, Oka et al. found that the slope of habitable land is positively associated with the incidence rate of hypertension based on the data from the nationwide medical checkups in Japan [35]. The village with the highest standardized mortality ratio of suicide had the highest slope of habitable land (15.4°) [36]. By contrast, a hilly environment was reported to be negatively associated with diabetes [19,20]. Fujiwara et al. found that an increase of one category (1.48°) in the interquartile range of the slope decreased the risk of poorly controlled diabetes mellitus by 18% among older adults [20]. Villanueva et al. calculated a neighborhood mean slope in metropolitan Perth, Australia [19]. Compared with adults living in the lowest levels of the slope, adults living in the moderate and higher levels were negatively associated with self-reported diabetes. A recent longitudinal study reported the relationship between slope (based on calculations using a GIS) and weight gain among older adults living in rural areas of Japan [29]. The slope degrees within the 400 m network buffer (mean slope) were positively associated with weight gain. Our results advance this debate, and further studies are needed to elucidate the potential pathways behind the association between land slope and health.

Several possible mechanisms underlie the association of land slope and depressive symptoms among older adults. A previous study reported that rural residents living in higher elevations are associated with knee pain [37]. Daily activities in a hilly environment may cause musculoskeletal pain through the mechanical load [17]. Musculoskeletal pain might be one of the causes of depressive symptoms [38]. In addition, it may be possible that the hilly environment discourages older adults with musculoskeletal pain from going for long walks [39]. As older adults with musculoskeletal pain had less physical activity and manifested more sedentary behavior, the lifestyle might cause depressive symptoms [33,40]. Conversely, several studies point out the positive influence of the undulating landform and hilly views on mental health. For example, a hilly environment tends to have beautiful scenery that may encourage older people to walk [41]. In addition, a previous study showed that a green landscape influences mental health and well-being in urban residents [42]. According to a systematic review, exposure to greenery, such as a forest, might positively affect general physical and mental health [43]. Further research is needed to confirm these associations and to explore the mechanisms in a rural area.

Our study showed the association between depressive symptoms with lower back pain and lack of sleep as individual factors. Our results support these associations reported in previous studies [9,10,44,45]. Among older residents living in a hilly environment, daily activities may cause musculoskeletal pain [17]. Occupational activities (e.g., climbing) appear to affect by causing osteoarthritis of the knees [46]. Musculoskeletal pain caused by osteoarthritis of the knees may contribute to sleep problems and depressive symptoms in older adults [10,18]. Thus, as lower back pain and sleep problems are two risk factors for depressive symptoms, it is important to address these factors in rural areas.

A few study limitations should be noted. First, the use of a cross-sectional design precludes any conclusions regarding causal relationships between environmental variables and depressive symptoms. Therefore, future observational longitudinal studies are needed. Second, the study sample included participants who completed annual health checkups across multiple centers within three municipalities. This could lead to a selection bias, as individuals who did not have the means to come to a center or lacked the motivation to do so were not sampled. As our study included only rural areas, our findings may have lower generalizability than those that include both urban and rural populations. Therefore, future studies should incorporate the urban population. In addition, the missing data could have led to an over- or underestimation of reported depressive symptoms. Third, the self-report nature of this study’s components using a questionnaire could be susceptible to participant response biases. Individuals with a severe mental illness may under- or overestimate their symptom profiles [12]. Finally, we could not account for the influence of any unmeasured variables that may affect the relationships between environmental variables and depressive symptoms, such as socio-economic status, living arrangement, social support, social activities, bereavement, poor health condition, lifestyle, prior depression, and subjective environmental variables [9,13,14].

## 5. Conclusions

The present study found that a hilly environment is associated with depressive symptoms in older adults in rural Japan. As individual factors, we showed the association between depressive symptoms with lower back pain and lack of sleep. Future longitudinal observational studies are required to investigate the causal association between the neighborhood environmental factors of land slope and depressive symptoms.

## Figures and Tables

**Table 1:** Participants’ characteristics.

Variables	Total	No Depressive Symptoms	Depressive Symptoms ^1^	*p*-Value ^2^
*n* (%)	935	720 (77.0)	215 (23.0)	
Sex				
Male	360 (38.5)	276 (76.7)	84 (23.3)	0.85
Female	575 (61.5)	444 (77.2)	131 (22.8)	
Age				
65–74 years old	718 (76.8)	552 (76.9)	166 (23.1)	0.87
≥75 years old	217 (23.2)	168 (77.4)	49 (22.6)	
Body mass index (Asian cut-off)				
Underweight, <18.5 kg/m^2^	69 (7.4)	51 (73.9)	18 (26.1)	0.44
Normal weight, 18.5–22.9 kg/m^2^	502 (53.7)	381 (75.9)	121 (24.1)	
Overweight, ≥23.0 kg/m^2^	364 (38.9)	288 (79.1)	76 (20.9)	
Current smoking				
No	874 (93.5)	675 (77.2)	199 (22.8)	0.54
Yes	61 (6.5)	45 (73.8)	16 (26.2)	
Current alcohol drinking				
No	505 (54.0)	389 (77.0)	116 (23.0)	0.99
Yes	430 (46.0)	331 (77.0)	99 (23.0)	
Physical activity				
≥150 min/week	795 (85.0)	615 (77.4)	180 (22.6)	0.54
<150 min/week	140 (15.0)	105 (75.0)	35 (25.0)	
Sedentary time				
<3 h/day	388 (41.5)	297 (76.5)	91 (23.5)	0.78
≥3 h/day	547 (58.5)	423 (77.3)	124 (22.7)	
Getting enough sleep				
Yes	749 (80.1)	620 (82.8)	129 (17.2)	**<0.01**
No	186 (19.9)	100 (53.8)	86 (46.2)	
Lower back pain				
No	488 (52.2)	401 (82.2)	87 (17.8)	**<0.01**
Yes	447 (47.8)	319 (71.4)	128 (28.6)	
Educational years				
≥12 years	375 (40.1)	284 (75.7)	91 (24.3)	0.45
<12 years	560 (59.9)	436 (77.9)	124 (22.1)	
Residential area				
Okinoshima town	114 (12.2)	92 (80.7)	22 (19.3)	0.06
Unnan city	365 (39.0)	292 (80.0)	73 (20.0)	
Ohnan town	456 (48.8)	336 (73.7)	120 (26.3)	
Land slope, degree, median (IQR)	9.19 (5.89, 12.65)	8.84 (5.71, 12.47)	10.49 (6.50, 16.94)	**0.01**

IQR, interquartile range; ^1^ depressive symptoms were defined by the cut-off point (>40 points) of the Zung Self-Rating Depression Scale; ^2^ statistical significance of the differences between groups (with and without depressive symptoms) was determined using the χ^2^-test for categorical data, and the Mann–Whitney U test for continuous data. Values in boldface show significance (*p* < 0.05).

**Table 2:** Associations of land slope with depressive symptoms in older adults.

		Crude Model			Adjusted Model		
		OR	95% CI	*p*-Value	OR	95% CI	*p*-Value
Land slope		**1.04**	**(1.01, 1.08)**	**0.01**	**1.04**	**(1.01, 1.08)**	**0.02**
Sex	Male				1	(reference)	
	Female				0.88	(0.60, 1.31)	0.54
Age	65–74 years old				1	(reference)	
	≥75 years old				1.55	(0.95, 2.52)	0.08
Body mass index (Asian cut-off)	Underweight, <18.5 kg/m^2^				1.19	(0.65, 2.20)	0.58
	Normal weight, 18.5–22.9 kg/m^2^				1	(reference)	
	Overweight, ≥23.0 kg/m^2^				0.81	(0.57, 1.15)	0.24
Current smoking	No				1	(reference)	
	Yes				1.21	(0.64, 2.31)	0.56
Current alcohol drinking	No				1	(reference)	
	Yes				0.91	(0.62, 1.33)	0.63
Physical activity	≥150 min/week				1	(reference)	
	<150 min/week				1.01	(0.64, 1.59)	0.96
Sedentary time	<3 h/day				1	(reference)	
	≥3 h/day				0.95	(0.68, 1.32)	0.74
Getting enough sleep	Yes				1	(reference)	
	No				**4.24**	**(2.94, 6.13)**	**<0.01**
Lower back pain	No				1	(reference)	
	Yes				**1.66**	**(1.19, 2.30)**	**<0.01**
Educational years	≥12 years				1	(reference)	
	<12 years				0.82	(0.59, 1.14)	0.24
Residential area	Okinoshima				1	(reference)	
	Unnan				0.75	(0.42, 1.35)	0.34
	Ohnan				1.44	(0.78, 2.66)	0.25
Cox and Snell R Square		0.007			0.094		
Nagelkerke R Square		0.011			0.142		
Hosmer–Lemeshow test, *p*-value		0.790			0.293		

OR, odds ratio; CI, confidence interval. Values in boldface show significance (*p* < 0.05).

## Data Availability

This study used data from the Shimane CoHRE study. Some of the data are available at the Center for Community-Based Healthcare Research and Education (CoHRE), Organization for Research and Academic Information, Shimane University, 223-8 Enya-cho, Izumo-shi, Shimane 693-8501, Japan.
